# Polystyrene and low-density polyethylene pellets are less effective in arsenic adsorption than uncontaminated river sediment

**DOI:** 10.1007/s11356-023-29218-w

**Published:** 2023-08-10

**Authors:** Thanh Kien Nguyen, Xiaowei Li, Lei Ren, Yuhan Huang, John L. Zhou

**Affiliations:** 1 Centre for Green Technology, School of Civil and Environmental Engineering, University of Technology Sydney, 15 Broadway, Sydney, NSW 2007 Australia; 2 Water Resources Division, Department of Environment, Parks and Water Security, Darwin, NT Australia; 3 School of Environmental and Chemical Engineering, Ministry of Education, Organic Compound Pollution Control Engineering, Shanghai University, Shanghai, 200444 People’s Republic of China; 4 College of Coastal Agricultural Sciences, Guangdong Ocean University, Zhanjiang, 524088 People’s Republic of China

**Keywords:** Adsorption, Arsenic, Kinetics, Microplastics, River sediment, Surface functional groups

## Abstract

The adsorption process of inorganic arsenic (As) plays an important role in its mobility, bioavailability, and toxicity in the river environment. In this work, the adsorption of dissolved arsenite (As(III)) and arsenate (As(V)) by microplastics (MPs) pellets (polystyrene (PS) and low-density polyethylene (LDPE)), river sediment, and their mixture were investigated to assess the adsorption affinities and mechanism. The adsorption kinetics showed slow and mild rising zones from the natural behavior of the chemical adsorption. The results indicated that both MP characteristics and water properties played a significant role in the adsorption behavior of inorganic As species. The As adsorption equilibrium was modeled well by both Langmuir and Freundlich isotherms and partly fitted with the Sips model suggesting that both mono-layer and multi-layer adsorption occurred during adsorption The spontaneous adsorption process for both As(III) and As(V) was evidenced by the adsorption thermodynamics. The maximum adsorption capacities of As(III) and As(V) reached 143.3 mg/kg and 109.8 mg/kg on PS in deionized water, which were higher than those on sediment-PS mixture (119.3 mg/kg, 99.2 mg/kg), which were all lower than on sediment alone (263.3 mg/kg, 398.7 mg/kg). The Fourier transform infrared spectroscopy analysis identified that As(III) and As(V) interaction with sediment surface functional groups was the main adsorption mechanism from surface complexation and coordination. Two functional groups of polystyrene (-NH_2_, -OH) were mainly involved in the adsorption of inorganic As species on PS, while -COO- and -OH functional groups contributed to the adsorption mechanism of inorganic As species on LDPE. The findings provide valuable insight on the adsorption behavior and mechanisms of As(III) and As(V) in river systems in the presence of MPs particles. Both PS and LDPE were shown to be less effective than river sediment in the adsorption of As species from water, which provides a different perspective in understanding the scale of MPs impact in pollutant transport in the aquatic environment.

**Graphical Abstract:**

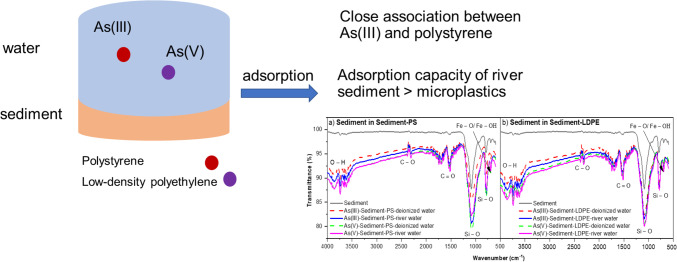

## Introduction

Microplastic particles (MPs) are plastic polymers with a diameter smaller than 5 mm (Dong et al. 2020; He et al. 2020) and have received wide concern due to their persistence in the environment and potential ecological effects (Shen et al. 2021). Polyethylene (PE) and polystyrene (PS) are the most popular plastic products and widely detected contaminants in fluvial environment. The density of PE varies from 0.917 to 0.965 g/cm^3^, while the density of PS (1.04–1.11 g/cm^3^) is slightly higher than that of fresh water (Zhou et al. 2020). Studies found that PE accounted for a high proportion of total MPs concentrations in river water and sediments. For example, it is estimated that PE accounts for 38 to 80% of total MPs in river water (Campanale et al. 2020; Lahens et al. 2018; Scherer et al. 2020; Zhang et al. 2015), and slightly lower proportion in river sediments, varying from 27 to 61% in Portugal, Germany, and China (Huang et al. 2021; Lin et al. 2018; Liu et al. 2021; Rao et al. 2020; Rodrigues et al. 2018; Scherer et al. 2020; Wang et al. 2017). Moreover, high PS abundance in water (20–34%) was reported in some Chinese rivers (Huang et al. 2021; Li et al. 2021; Wu et al. 2020; Zhou et al. 2020). Studies revealed that different MPs can adsorb pollutants such as antibiotics (Li et al. 2018), perfluorooctanoic sulphonamide (Wang et al. 2015), and heavy metals (Holmes et al. 2012; Li et al. 2019). The adsorption of heavy metals such as Cu, Cd, Cr, Pb, As, Zn, Ni, and Co on MPs has been investigated to understand potential capturing of metals, adsorption behavior, and mechanisms by MPs, specifically PE and PS (Dong et al. 2020; Godoy et al. 2019; Wang et al. 2020; Zhang et al. 2020; Zong et al. 2021). Godoy et al. (2019) suggested that chemical adsorption was the main adsorption mechanism due to better description of experimental data by the Langmuir model than by the Freundlich model for several types of MPs including PE, polyethylene terephthalate (PET), polypropylene (PP), polystyrene (PS), and polyvinyl chloride (PVC).

Sediment is a vital compartment in river systems and functions as a sink of heavy metals, MPs, and other pollutants such as pharmaceuticals (Jahan and Strezov 2018; Nematollahi et al. 2021). Among metals and metalloids contaminated in environments, arsenic (As) is a highly toxic element which is present in natural water, sediments, and biota (Chen et al. 2016; Goldberg and Suarez 2013; Osuna-Martínez et al. 2021). Natural processes (i.e., weathering and biological activity) and anthropogenic activities (e.g., mining, industrial processes, and agricultural activities) have been reported as the main sources of As releasing into the environment (Hua 2018; Osuna-Martínez et al. 2021; Xie et al. 2018). In terrestrial and aquatic environments, As predominantly occurs as As(III) and As(V), which are more toxic than organic arsenic forms (Wang et al. 2018; Xie et al. 2018). In surface water such as river water, As pollution is considered a major environmental problem (Hua 2018). Sediments can release high As concentrations to water under the changes in physical and chemical factors and favorable hydraulic conditions (Chen et al. 2016; Jahan and Strezov 2018; Nematollahi et al. 2021). The sedimentary redox conditions and sediment properties (e.g., grain size, organic matter content) influenced the chemical speciation, concentration, and distribution of As in sediment (Wang et al. 2019), resulting in various degrees of As bioavailability and toxicity (Ma et al. 2015). As a result, contaminated sediments are a secondary As pollution source (Chen et al. 2016; Nematollahi et al. 2021). Thus, studying the transport and fate of As in the sediment and aqueous environments is a critical element of environmental quality assessment (Wang et al. 2018).

Adsorption studies have been widely conducted for evaluating the fate and behavior of As in soils and sediments, as well as their components such as Al and Fe oxides, clay minerals, organic matter, particle size fractions, and natural humic materials (Dousova et al. 2012; Goldberg and Suarez 2013; Li et al. 2018; Wang and Mulligan 2006; Yang et al. 2006; Zhang and Selim 2005). Other controlling factors such as pH, competitive anions and cations, bacterial activity, concentrations of As in solution, reaction time, and adsorbent dosages have been studied to explore their effects on the adsorption capacities of soils and sediments (Huang et al. 2013; Xie et al. 2018). Adsorption thermodynamics were used to evaluate the relationship between the heat of adsorption and adsorption mechanism (Rupam et al. 2020). The application of Gibbs free change (Δ*G*), entropy change (Δ*S*^o^), and enthalpy change (Δ*H*^o^) as a function of temperature, pressure, and adsorbate uptake indicated that adsorption of phenol on magnetic carbon nanotube materials was endothermic (Lin et al. 2021a), while exothermic mechanism occurred for Ni^2+^ and Pb^2+^ (Hu and Zou 2023; Lin et al. 2021b). In addition, all these adsorption processes were spontaneous.

The interactions between arsenic and MPs are of high importance as both are ubiquitous in the environment. Dong et al. (2019; 2020) investigated the adsorption of As(III) onto different MPs and reported that high adsorbed amount of As(III) onto polytetrafluoroethylene (PTFE) and PS MP particles was in accordance with large specific surface area (SSA) of the particles, low pH solution values, and low concentration of interfering nitrate and phosphate ions in the solution. Moreover, As(III) adsorption on PTFE and PS was not significant at pH 3 and 4 and then decreased gradually when pH was increased from 4 to 7. This process was explained by the change of OH^−^ content related to pH in solution. Low OH^−^ content at low solution pH (3–4), covering the levels of the point zero charged (PZC) of PTFE and PS, does not compete with arsenic anion during the adsorption process. When the solution pH exceeded 4, these adsorbents became negatively charged and repulsed arsenate ions, leading to the reduction of As(III) adsorption. These studies also found that the presence of NO_3_^−^ and PO_4_^3−^ in the solution inhibited the adsorption of As(III) onto PTFE and PS, in which higher concentrations of these anions caused a decrease in As(III) adsorption.

Studies on the kinetic and equilibrium adsorption of As onto different types of MPs have been conducted under laboratory conditions (Dong et al. 2020, 2019). Controlling factors including pH, temperature, and interfering NO_3_^−^ and PO_4_^3−^ ions influencing As(III) adsorption on polytetrafluoroethylene (PTFE) and PS were reported. The SSA, pH, NO_3_^−^ and PO_4_^3−^ showed similar effects on adsorption of As(III) on MPs as on soils. The polarity, morphology, and organic polymer composition also enhanced heavy metal adsorption on MPs (Ahmed et al. 2021; Ashton et al. 2010). The adsorption of As(III) on the surface of MPs primarily occurs via hydrogen bonds of carboxyl groups, while electrostatics and non-covalent forces are the main interactions mechanisms of adsorption (Dong et al. 2020). However, questions remain on how As contaminants behave in river systems with the presence of both MPs and sediment.

Therefore, this study aims to firstly investigate the adsorption kinetics and equilibrium of As(III)/As(V) species on MPs with or without the presence of river sediment in deionized water and river water; to study the adsorption mechanism of As(III) and As(V) related to surface functional groups of MPs and river sediment; and to explore the structure and properties of PS and LDPE pellets by Fourier transform infrared (FTIR) spectroscopy before and after adsorption of As(III) and As(V).

## Materials and methods

### Chemical standards

PS and LDPE resin pellets (3 mm in particle size) purchased from Sigma-Aldrich Australia were used as the adsorbents. Multi-element standard solution 4 for ICP (40 mg/L of As) was supplied by Sigma-Aldrich Pty Ltd., Australia. The solution was diluted by deionized water with a resistivity of 18 MΩ. Analytical grade sodium arsenate dibasic heptahydrate (Na_2_HAsO_4_⋅7H_2_0) and sodium arsenite (NaAsO_2_), purchased from Sigma-Aldrich Pty Ltd, were dissolved with deionized water to obtain As(III) and As(V) stock solutions (100 mg/L). The phosphoric acid (H_3_PO_4_, 85% w/w) and hydroxylamine hydrochloride (NH_2_OH⋅HCl, 99% purity) were also obtained from Sigma-Aldrich Pty Ltd. Then, solutions of 1.0 M phosphoric acid and 0.2 M hydroxylamine hydrochloride were prepared by diluting their original standard solution with deionized water. All plasticware and glassware were soaked in 2% (v/v) HNO_3_, followed by repeated rinsing with deionized water, and then dried before use.

### Field sampling

River sediments from Bargo Riverbank, New South Wales, Australia, have been reported to be unpolluted to moderately polluted with heavy metals due to mining activity, while Bargo River water quality was determined as slightly polluted (Ali et al. 2018; Nguyen et al. 2022). The river water was used as feeding solution providing real environmental characteristics in comparison with deionized water. Bargo River sediment was chosen as natural adsorbent for As sorption experiments. After sampling, the sediment samples were packed in sealed polyethylene bags, and then stored in a cool box at 4 °C when transferred to the laboratory. The sediment samples were dried in an oven at 105 °C for 24 h to remove moisture content and then passed through 2-mm stainless steel sieve before further analysis. The Bargo River water and sediment characteristics were reported by Nguyen et al. (2022).

### Adsorption experiments

The adsorption kinetics and isotherm experiments of As(III)/As(V) adsorption onto PS and LDPE were performed in triplicate. The stock solution of 100 mg/L of As(III)/As(V) was diluted by deionized water or river water to obtain a range of initial As(III) or As(V) solutions (0.5, 2.0, 8.0, and 10 mg/L). A mixture of approximately 2.0 g PS/LDPE and 200 mL diluted As solution (10.0 mg/L) was placed in 500-mL beaker which was placed on a horizontal shaker at 120 rpm for 7 days. In addition, the adsorption of As(III)/As(V) was also conducted by mixing 2.0 g of PS/LDPE and 2.0 g of river sediment in 200 mL of 10.0 mg/L As(III)/As(V) in deionized water or river water for 7 days. During the adsorption process, 3 mL suspension samples were taken at regular intervals (3, 6, 12, 24, 72, 120, and 168 h), filtered by a 0.45-μm membrane filter, and analyzed for total As by ICP-MS (Agilent 7900). The adsorption of As(III) or As(V) was evaluated by monitoring the decrease of arsenic concentration in solution.

### Sediment characterization

Bargo River sediment samples were ground to fine powder before characterization for important properties. Its organic matter content (0.4%) was determined by the loss-on-ignition method. The SSA (4.6 m^2^/g) was determined by the BET isotherm method from N_2_ adsorption. The metal contents in sediment were determined by acid extraction followed by ICP-MS (Agilent 7900) analysis, for Al (33.7 mg/kg), Fe (36.7 mg/kg), As (0.2 mg/kg), Cr (0.08 mg/kg), Zn (1.8 mg/kg), Cd (0.06 mg/kg), and Pb (0.2 mg/kg) (Nguyen et al. 2022). As a result, this river sediment is defined as the uncontaminated adsorbent for experiments in this study. The FTIR spectra (500–4000 cm^−1^) of river sediment, PS, and LDPE pellets before and after the adsorption of As(III) and As(V) were analyzed by using Shimadzu MIRacle 10 (Japan) in order to investigate the surface sorption mechanisms.

### Kinetic and equilibrium sorption studies

Arsenic concentrations retained in the adsorbent phase (mg/kg) are calculated by Eqs. (1) and (2):

1 $${\mathrm{q}}_{\mathrm{t}}=\frac{{(\mathrm{C}}_{\mathrm{o}}-{\mathrm{C}}_{\mathrm{t}})\mathrm{V}}{\mathrm{m}}$$

2 $${\mathrm{q}}_{\mathrm{e}}=\frac{{(\mathrm{C}}_{\mathrm{o}}-{\mathrm{C}}_{\mathrm{e}})\mathrm{V}}{\mathrm{m}}$$

where *q*_*t*_ (mg/kg) and *C*_*t*_ (mg/L) are the concentrations of As(III) or As(V) in adsorbent and solution at time *t*, *q*_*e*_ (mg/kg) and* C*_*e*_ (mg/L) are the concentrations of As(III) or As(V) in adsorbent and solution at equilibrium, *C*_*o*_ (mg/L) is As(III) or As(V) concentration at the initial time, *V* (mL) is the volume of solution, and *m* is the mass of adsorbent (g).

The PFO and PSO equations have been widely applied to assess the adsorption kinetics of As on soils, sediments, and MPs (Dong et al. 2020; Gedik et al. 2016; Guo et al. 2007; Kumar et al. 2016; Kundu and Gupta 2006; Luo et al. 2019; Ma et al. 2015; Rawat et al. 2022). The PFO model can well describe the initial adsorption stage (Ho and McKay 1999; Ma et al. 2015), while the PSO model was better for describing the physical or chemical adsorption at a site (Kumar et al. 2016) or the whole adsorption process (Ma et al. 2015).

The adsorption experimental data was analyzed by PFO and PSO models using non-linear regression equation (Alkurdi et al. 2021; Ma et al. 2015), as shown in Eqs. (3) and (4), respectively. The linear forms of PFO and PSO kinetic models are straightforward for application; however, the erroneous values of calculating kinetic parameters were the drawbacks (Nguyen et al. 2022). In contrast, the non-linear forms provided better results in comparison to linear regression analysis (Rawat et al. 2022). Thus, the non-linear forms of PFO and PSO models for the adsorption kinetics are used in this study (Ma et al. 2015):

3 $${\mathrm{q}}_{\mathrm{t}}={\mathrm{q}}_{\mathrm{e}}(1-{\mathrm{e}}^{-{\mathrm{k}}_{1}\mathrm{t}})$$

4 $${\mathrm{q}}_{\mathrm{t}}=\frac{{\mathrm{q}}_{\mathrm{e}}^{2}{\mathrm{k}}_{2}\mathrm{t}}{1+{\mathrm{q}}_{\mathrm{e}}{\mathrm{k}}_{2}\mathrm{t}}$$

Another model commonly applied for simulating the kinetics of adsorption is Elovich equation, which was developed by López-Luna et al. (2019) and Plazinski et al. (2009) as below:

5 $${\mathrm{q}}_{\mathrm{t}}=\frac{1}{\upbeta }\mathrm{ln}\left(1+\mathrm{\alpha \beta t}\right)$$

where *q*_*t*_ is the amount (mg/kg) of As(III) or As(V) adsorbed at time *t*, *q*_*e*_ is the amount (mg/kg) of As(III) or As(V) at the equilibrium, *k*_*1*_ (1/h) is the equilibrium rate constant of the PFO model, *k*_*2*_ (kg/mg-h) is the equilibrium rate constant of the PSO model, α is the Elovich initial adsorption rate (mg/kg min), and β is desorption constant (kg/mg).

The approaching equilibrium parameter of Elovich equation is defined by Wu et al. (2009) as:

R_E_ = 1/(q_e_ x β) to classify the characteristic curves of kinetic adsorption.

The adsorption equilibrium at the interface between adsorbent and liquid phases is widely interpreted by the Langmuir and Freundlich adsorption isotherms (Tseng et al. 2009). The Langmuir isotherm model (Eq. (6)) is used to describe the mono-molecular layer adsorption (Rawat et al. 2022):

6 $${\mathrm{q}}_{\mathrm{e}}= \frac{{\mathrm{K}}_{\mathrm{L}}{\mathrm{q}}_{\mathrm{m}}{\mathrm{C}}_{\mathrm{e}}}{1+{\mathrm{K}}_{\mathrm{L}}{\mathrm{C}}_{\mathrm{e}}}$$

where *K*_*L*_ represents the bonding energy constant (mg/L) and *q*_*m*_ is the maximum adsorption capacity (mg/kg).

Additionally, the equilibrium parameter (*R*_*L*_) is used to explain the essentiality of Langmuir adsorption isotherms by Eq. (7) (Rawat et al., 2021):

7 $${\mathrm{R}}_{\mathrm{L}}= \frac{1}{1+{\mathrm{K}}_{\mathrm{L}}{\mathrm{C}}_{\mathrm{e}}}$$

where the values of *R*_*L*_ > *1*, *0* < *R*_*L*_ < *1*, *R*_*L*_ = *1*, and *R*_*L*_ = *0* indicate unfavorable, favorable, linear and irreversible adsorption of As(III) and As(V) on the surface of sediment particles, respectively (Rawat et al. 2022).

The Freundlich sorption model (Eq. (8)) is an empirical adsorption equation indicating that adsorption sites on the surface of adsorbent possess different adsorption energies (Wang et al. 2018):

8 $${\mathrm{q}}_{\mathrm{e}}={\mathrm{K}}_{\mathrm{F}} \times {\mathrm{C}}_{\mathrm{e}}^{1/\mathrm{n}}$$

where *K*_*F*_ is the Freundlich constant or capacity factor (mg/kg-(L/mg)^n^), while 1/*n* is the Freundlich exponent.

A three-parameter isotherm model, Sips, is formed by the combination from Langmuir and Freundlich expressions (Alkurdi et al. 2021). This model reduces to the Freundlich model at the low adsorbate concentrations or to the Langmuir model, while the adsorbate concentrations are high (Foo and Hameed 2010).

9 $${\mathrm{q}}_{\mathrm{e}}=\frac{{\mathrm{q}}_{\mathrm{m}}\mathrm{ x }{(\mathrm{K}}_{\mathrm{s }}\mathrm{x }{\mathrm{C}}_{\mathrm{e}})^{\mathrm{n}}_{\mathrm{s}}}{\left(1+{(\mathrm{K}}_{\mathrm{s}}{\mathrm{ x C}}_{\mathrm{e}})^{\mathrm{n}}_{\mathrm{s}}\right)}$$

where *K*_*s*_ is the Sips constant related to the energy of adsorption process and *n*_*s*_ is the exponential factor of the isotherm.

### Adsorption thermodynamics

The Gibbs free energy change (Δ*G*), an important parameters in thermodynamics to provide information about the energy change and adsorption mechanism during the adsorption process, is calculated by Hu and Zou (2023) as:

10 $$\Delta \mathrm{G}=\mathrm{R x T x In}\left({\mathrm{K}}_{\mathrm{e}}\right)$$

where *R* (2078.5 J kg^−1^ K^−1^) is the gas constant, *T* (68 K) is the temperature in Kelvin (20 °C), and *K*_*e*_ = *q*_*e*_*/C*_*e*_ at the equilibrium.

## Results and discussion

### Adsorption kinetics

#### Adsorption kinetics of As(III)/As(V) by PS or LDPE pellets

**Fig. 1:**
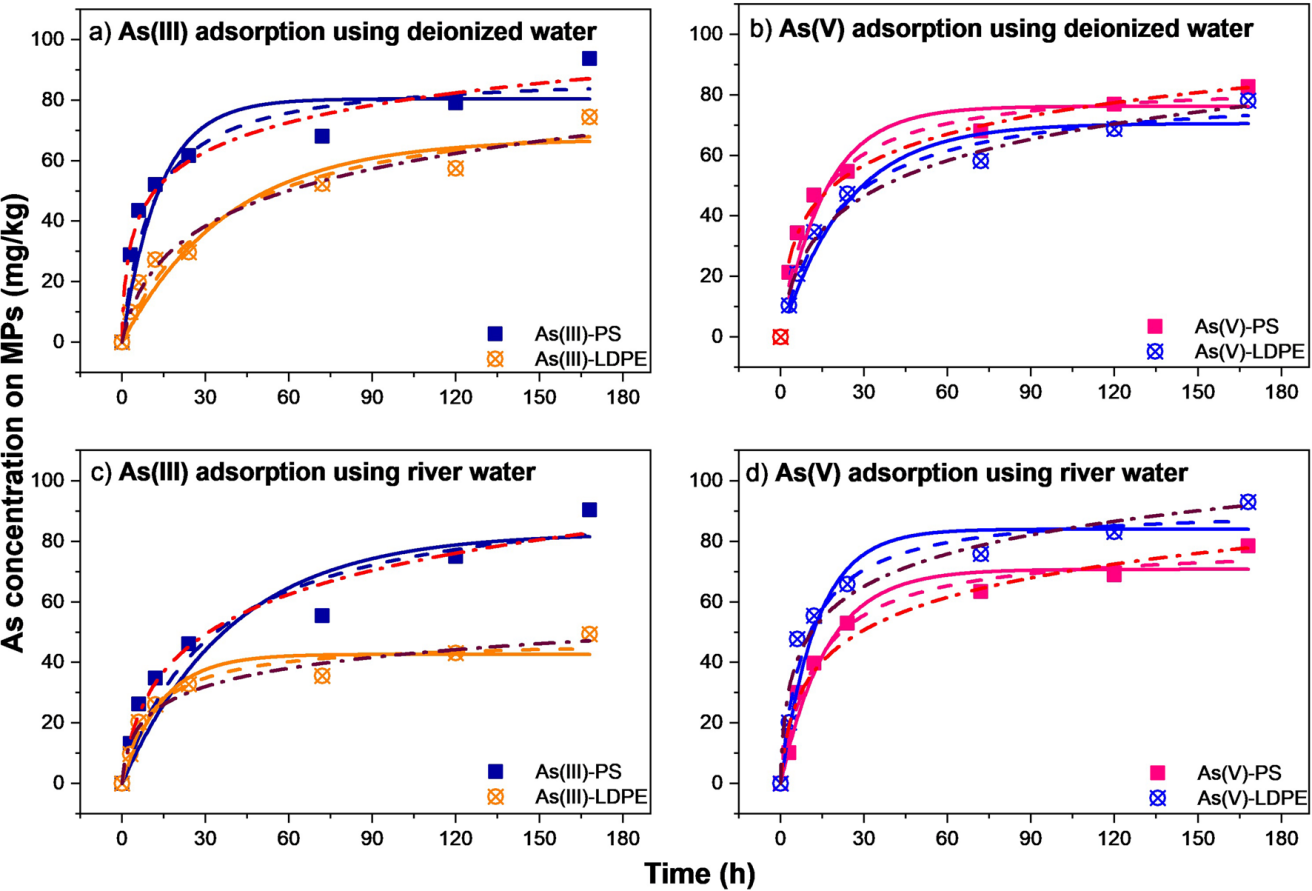
The PFO and PSO kinetic models of As(III) adsorption in PS and LDPE pellets (**a** and **c**) and As(V) adsorption on PS and LDPE pellets (**b** and **d**). The solid, dash, and dash dot lines represent the non-linear fitting by PFO, PSO, and Elovich models, respectively

The results of As(III) and As(V) adsorption by PS and LDPE adsorbents showed an initial fast stage of adsorption up to 24 h, followed by a slower stage (Fig. 1). Table 1 illustrates the fitting results of PFO and PSO models for As(III) and As(V) adsorption on PS and LDPE pellets. Studies by Dong et al. (2019; 2020) pointed out that the adsorption rate of As(III) on PTFE and PS slowly increased during the first 2 h, followed by rapidly increasing up to 60 h for PTFE and 40 h for PS, respectively. The first adsorption phase was explained the rapid occupation by As(III)/As(V) on external surface adsorption sites of these MPs, and then, As species entered the adsorption sites in the inner surface (Dong et al. 2020). Generally, PS particles showed higher adsorption capacities than LDPE for As(III)/As(V) (Fig. 1a–c), except for As(V) in river water (Fig. 1d).

**Table 1:** The process parameters for the adsorption kinetics and equilibrium of As(III)/As(V)

As	Experimental condition	PFO			PSO			Elovich			*q*_*e*_^c^ (mg/kg)	Reference
		*R*^2^	*K*_1_ (1/h)	*q*_*e*_^a^ (mg/kg)	*R*^2^	*K*_2_ (kg/mg-h)	*q*_*e*_^b^ (mg/kg)	*R*^2^	α (mg/kg h)	β (kg/mg)		
As(III)	PS-deionized water	0.989	0.074	86.5	0.994	0.0011	89.0	0.979	39.92	0.07	93.8	This study
	LDPE-deionized water	0.994	0.026	67.2	0.996	0.0003	82.5	0.976	4.27	0.05	74.5	This study
	PS-river water	0.988	0.025	82.7	0.992	0.0003	98.4	0.971	7.01	0.05	90.4	This study
	LDPE-river water	0.997	0.071	42.7	0.998	0.0020	47.5	0974	10.53	0.11	49.3	This study
	PS (0.1–1 µm)	0.908		1154	0.987		2094				1165	Dong et al. (2020)
	PS (1–10 µm)	0.602		1110	0.899		2013				1115	Dong et al. (2020)
	PS (> 10 µm)	0.771		1033	0.957		2043				1035	Dong et al. (2020)
	Sediment-PS-deionized water	0.993	0.08	77.1	0.996	0001	84.3	0.974	34.44	0.07	88.6	This study
	Sediment-LDPE-deionized water	0.993	0.07	76.6	0.997	0.001	85.1	0.991	955.54	0.12	86.4	This study
	Sediment-deionized water	0.987	0.06	91.7	0.992	0.001	103.5				105.6	Nguyen et al. (2022)
	Sediment-PS-river water	0.996	0.13	76.2	0.998	0.003	79.8	0.989	25.59	0.07	82.7	This study
	Sediment-LDPE-river water	0.994	0.07	67.5	0.997	0.001	74.4	0.983	27.31	0.08	76.3	This study
	Sediment-river water	0.995	0.04	77.3	0.997	0.001	90.4				85.8	Nguyen et al. (2022)
As(V)	PS-deionized water	0.995	0.062	76.2	0.998	0.0010	84.6	0.990	21.12	0.07	82.7	This study
	LDPE-deionized water	0.994	0.042	70.5	0.998	0.0006	81.8	0.984	7.37	0.06	78.0	This study
	PS-river water	0.997	0.060	70.8	0.999	0.0010	79.0	0.975	12.06	0.06	78.5	This study
	LDPE-river water	0.995	0.077	84.0	0.998	0.0013	91.3	0.968	33.01	0.06	93.1	This study
	Sediment-PS-deionized water	0.996	0.06	64.5	0.998	0.001	72.5	0.994	15.30	0.08	72.0	This study
	Sediment-LDPE-deionized water	0.995	0.04	73.9	0.998	0.001	86.6	0.968	421.89	0.10	81.2	This study
	Sediment-deionized water	0.989	0.06	115.6	0.995	0.001	173.5				168.6	Nguyen et al. (2022)
	Sediment-PS-river water	0.997	0.14	82.1	0.998	0.004	85.5	0.991	6.95	0.05	89.2	This study
	Sediment-LDPE-river water	0.995	0.09	84.4	0.998	0.002	90.5	0.974	53.88	0.07	93.3	This study
	Sediment-river water	0.989	0.08	82.6	0.993	0.001	89.8				94.3	Nguyen et al. (2022)

^a,b^Estimated equilibrium adsorption capacity from the PFO and PSO models

^c^Equilibrium adsorption capacity from the adsorption isotherms

More specifically, the adsorption capacities of PS pellets calculated from the experimental data at equilibrium in deionized water were higher than in river water for both As(III) and As(V) (Table 1), at 93.8 mg/kg and 90.4 mg/kg for As(III), and 82.7 mg/kg and 78.5 mg/kg for As(V), respectively. Similar trend was found for the adsorption of As(III) on LDPE pellets, at 74.5 mg/kg in deionized water compared to 49.3 mg/kg in river water. However, the adsorption of As(V) on LDPE pellets was higher in river water (93.1 mg/kg) than in deionized water (78.5 mg/kg). The amount of As(III) adsorbed on PS pellets in this study was significantly lower than that from the study of Dong et al. (2020), which varied between 1035 and 1165 mg/kg for different sizes of PS particles. The correlation coefficient (*R*^2^) for the adsorption of As(III) and As(V) on PS and LDPE was high (between 0.988 and 0.997), indicating that PFO modeled well with the experimental data. These *R*^2^ values from the non-linear PFO model were higher than those calculated from the linear regression for As(III) adsorption on PS particles (Dong et al. 2020), supporting that the non-linear form would be better for the description of adsorption kinetics (Rawat et al. 2022). In addition, the estimated adsorption capacities of these MP pellets at the equilibrium from PFO model were lower than those calculated from the experimental data (Table 1). The PFO *q*_*e*_ values for As(III) were 86.5 mg/kg (PS-deionized water), 67.2 mg/kg (LDPE-deionized water), 82.7 mg/kg (PS-river water), and 42.7 mg/kg (LDPE-river water), while the values for As(V) were 76.2 mg/kg (PS-deionized water), 70.5 mg/kg (LDPE-deionized water), 70.8 mg/kg (PS-river water), and 84.0 mg/kg (LDPE-river water).

Additionally, the approaching equilibrium parameter (*R*_*E*_), which is derived from the Elovich equation, is presented in Table 1. The results of *R*_*E*_ showed that the kinetic adsorption of both As(III) and As(V) on PS and LDPE occurred at mild rising zone of the chemical adsorption where the *R*_*E*_ values were between 0.1 and 0.3 (Wu et al. 2009).

The amount of As(III) and As(V) adsorption on PS pellets and LDPE pellets estimated by PSO model was closer to the experimental value than that from PFO (Table 1), which was opposite to the results estimated by linear regression (Dong et al. 2020). Azizian (2004) and Rawat et al. (2022) stated that non-linear forms of kinetic adsorption (PFO and PSO) generated better results than the linear forms. Our results supported those suggestions and confirmed the role of PSO model in describing physicochemical mechanism during As adsorption process (Kumar et al. 2016; Ma et al. 2015).

#### Adsorption kinetics of As(III)/As(V) in river sediments with PS or LDPE pellets

**Fig. 2:**
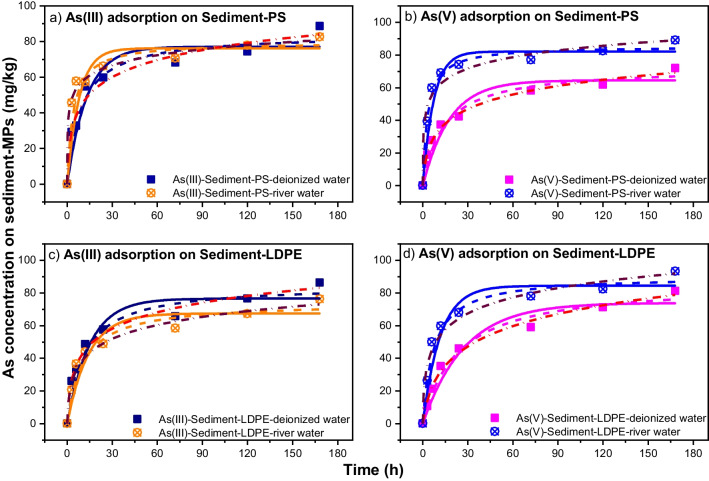
PFO and PSO kinetic model of As(III) adsorption by sediment-PS and sediment-LDPE pellets (**a** and **c**) and As(V) adsorption by sediment-PS and sediment-LDPE pellets (**b** and **d**). The solid, dash, and dash dot curves depict results of curve-fitting with the PFO, PSO, and Elovich models, respectively

The kinetic experimental result was simulated by PFO and PSO models to illustrate the changes in the adsorbed amounts of As(III) and As(V) versus time. The results of PFO and PSO models for As(III) and As(V) adsorption on sediment-PS and sediment-LDPE in deionized water or river water provided good fitting with experimental data (Fig. 2). The kinetic parameters of the PFO and PSO models for these experiments are presented in Table 1. The PSO model provided more similar results of q_e_ to the experimental data than PFO. In comparison, there was a higher adsorption affinity of As(III) in deionized water than in river water, while the opposite trend was observed for As(V). It was revealed that the chemisorption mechanism was favored for different conditions (Alkurdi et al. 2021). In contrast, the adsorption rates for the first adsorption phase of As(III) on sediment-PS and sediment-LDPE were higher than As(V) in both deionized water and river water. Compared with adsorption on PS/LDPE pellets only, higher adsorption capacities of As(III) in sediment-PS and sediment-LDPE than those in PS or LDPE only in river water, but opposite trend was observed in deionized water. For As(V), the adsorption capacities in sediment-PS or sediment-LDPE were higher than in PS or LDPE only, except the sediment-PS in deionized water.

Previously, the adsorption capacities of this sediment for As(III)/As(V) are studied (Nguyen et al. 2022), as shown in Table 1. In comparison, the amounts of As(III) and As(V) adsorbed on sediment/MPs mixture were lower than those adsorbed on the sediment alone at equilibrium. Using deionized water as an example, 105.6 mg/kg of As(III) was adsorbed in sediment alone, compared to 88.6 mg/g by sediment-PS and 86.4 mg/kg by sediment-LDPE. Similarly for As(V) at equilibrium, sediment adsorbed 168.6 mg/kg, higher than 72 mg/kg on sediment-PS and 81.2 mg/kg on sediment-LDPE. Such differences can be broadly explained by the lower adsorption by both PS and LDPE than by sediment (Table 1). The results appear to differ from previous reports (e.g., Dong et al. 2020) who reported over 10 times higher adsorption capacity of As(III) by PS.

The results for Elovich kinetic model indicated that adsorption of As(V) on sediment-PS and sediment-LDPE in river water occurred in slowing rising zone of natural chemical adsorption with *R*_*E*_ values > 0.3, while under other conditions, it showed the mild rising zone of chemical adsorption (Wu et al. 2009).

### Non-linear adsorption isotherms

#### Adsorption isotherms of As(III)/As(V) by PS or LDPE pellets

**Fig. 3:**
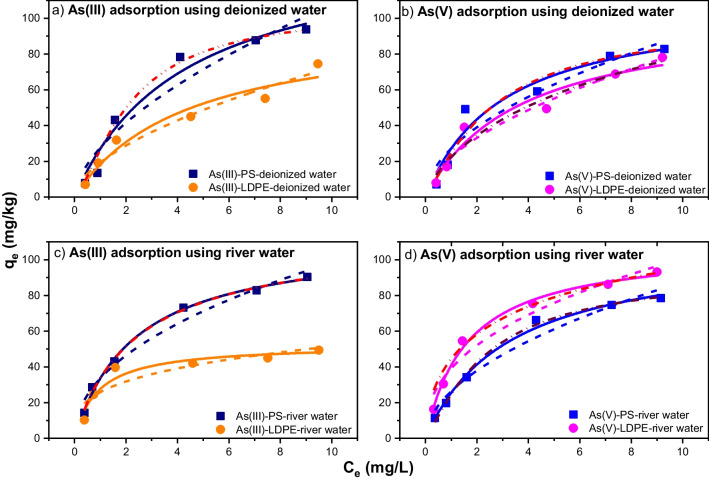
The adsorption isotherm of As(III) on PS and LDPE pellets (**a** and **c**) and As(V) on PS and LDPE pellets (**b** and **d**). The solid, dash, and dash dot curves depict results of curve-fitting with the Langmuir, Freundlich, and Sips equations, respectively

The adsorption isotherms of As(III)/As(V) for PS pellets and LDPE pellets by using deionized water and river water were modeled by the Langmuir and Freundlich adsorption equations (Fig. 3). It can be seen that both Langmuir and Freundlich equations fitted well to As(III) and As(V) adsorption isotherms which exhibited non-linear behavior. In addition, the best-fit parameter values (*q*_*m*_, *K*_*L*_, *K*_*F*_, *n*) and *R*^2^ for As(V) and As(III) at the equilibrium time are summarized in Table 2. The marginal differences among the values of *R*^2^ for both Langmuir and Freundlich models indicated that these models can well describe the adsorption isotherms for PS pellets and LDPE pellets related to As(III) and As(V). Previous studies showed that the Langmuir and Freundlich models were applied for various heavy metals, and the goodness of the fitting with the dataset was not very different between these models (Collard et al. 2019; Dong et al. 2020; Holmes et al. 2014; Hosseinpour et al. 2018).

**Table 2:** The Langmuir, Freundlich, and Sips equation parameters for As(III) and As(V) adsorption

	Langmuir			Freundlich			Sips							Reference
	*q*_*m*_ (mg/kg)	*K*_*L*_ (L/mg)	*R*^2^	*K*_*F*_	1/*n*	*R*^2^	*q*_*m*_ (mg/kg)	*K*_*s*_		*n*_*s*_		*R*^2^		
**As(III)**														
PS-deionized water	143.4 ± 24.3	0.23 ± 0.09	0.965	27.55 ± 6.41	0.59 ± 0.12	0.924	105.7 ± 6.6		0.46 ± 0.06		1.43 ± 0.35		1.000	This study
LDPE-deionized water	98.2 ± 18.6	0.23 ± 0.10	0.948	19.32 ± 3.00	0.57 ± 0.08	0.961	N/A							This study
PS-river water	112.4 ± 3.6	0.43 ± 0.04	0.996	33.61 ± 3.09	0.47 ± 0.05	0.975	115.1 ± 13.1		0.40 ± 0.12		0.97 ± 0.13		0.996	This study
LDPE-river water	52.7 ± 4.1	1.10 ± 0.33	0.928	25.88 ± 3.83	0.30 ± 0.08	0.824	N/A							This study
PS (0.1–1 µm)	1120	0.0006	0.951	0.017	0.887	0.875								Dong et al. (2020)
PS (1–10 µm)	1047	0.0013	0.904	0.017	0.903	0.901								Dong et al. (2020)
PS (> 10 µm)	920	0.0001	0.906	0.015	0.907	0.950								Dong et al. (2020)
Sediment-PS-deionized water	119.3 ± 29.0	0.27 ± 0.17	0.938	25.80 ± 5.51	0.56 ± 0.11	0.954	N/A							This study
Sediment-LDPE-deionized water	171.3 ± 105.1	0.10 ± 0.10	0.954	19.32 ± 5.46	0.66 ± 0.14	0.945	N/A							This study
Sediment-deionized water	263.3 ± 73.8	0.08 ± 0.03	0.986	21.5 ± 2.2	0.74 ± 0.10	0.991								Nguyen et al. (2022)
Sediment-PS-river water	112.0 ± 29.0	0.26 ± 0.14	0.946	22.67 ± 3.09	0.56 ± 0.07	0.981	N/A							This study
Sediment-LDPE-river water	97.1 ± 7.8	0.33 ± 0.08	0.990	23.99 ± 3.25	0.53 ± 0.07	0.979	104.1 ± 35.3		0.28 ± 0.24		0.94 ± 0.28		0.990	This study
Sediment-river water	234.3 ± 109.9	0.06 ± 0.05	1.000	16.8 ± 1.3	0.75 ± 0.19	1.000								Nguyen et al. (2022)
**As(V)**														
PS-deionized water	109.8 ± 17.0	0.33 ± 0.13	0.945	27.17 ± 5.64	0.52 ± 0.11	0.953	101.1 ± 26.9		0.39 ± 0.18		1.19 ± 0.74		1.000	This study
LDPE-deionized water	103.5 ± 17.1	0.27 ± 0.11	0.953	22.47 ± 3.72	0.56 ± 0.09	0.955	182.9 ± **345.3**		0.07 ± 0.31		0.73 ± 0.48		0.956	This study
PS-river water	108.9 ± 6.0	0.30 ± 0.04	0.993	26.43 ± 3.51	0.52 ± 0.07	0.964	94.6 ± 8.6		0.42 ± 0.09		1.21 ± 0.17		0.996	This study
LDPE-river water	107.7 ± 3.7	0.61 ± 0.07	0.993	39.23 ± 3.97	0.41 ± 0.06	0.958	165.6 ± 68.8		0.17 ± 0.25		0.56 ± 0.22		1.000	This study
PS (0.1–1 µm)														Dong et al. (2020)
PS (1–10 µm)														Dong et al. (2020)
PS (> 10 µm)														Dong et al. (2020)
Sediment-PS-deionized water	99.2 ± 5.5	0.32 ± 0.05	0.994	25.47 ± 4.39	0.50 ± 0.09	0.956	84.8 ± 4.19		0.44 ± 0.05		1.28 ± 0.11		0.998	This study
Sediment-LDPE-deionized water	94.7 ± 14.2	0.44 ± 0.21	0.937	28.47 ± 4.11	0.47 ± 0.07	0.962	N/A							This study
Sediment-deionized water	398.7 ± 90.0	0.09 ± 0.03	0.989	35.1 ± 4.2	0.75 ± 0.11	0.987								Nguyen et al. (2022)
Sediment-PS-river water	103.6 ± 10.1	0.45 ± 0.14	0.973	32.02 ± 4.91	0.45 ± 0.08	0.955	107.5 ± 39.0		0.41 ± 0.39		0.95 ± 0.44		0.973	This study
Sediment-LDPE-river water	107.9 ± 8.4	0.53 ± 0.14	0.978	37.68 ± 3.03	0.42 ± 0.04	0.984	171.9 ± 104.8		0.14 ± 0.26		0.64 ± 0.22		0.989	This study
Sediment-river water	206.2 ± 37.1	0.10 ± 0.03	1.000	21.2 ± 2.9	0.69 ± 0.14	1.000								Nguyen et al. (2022)

*N/A*, the Sips model is not applicable for this condition.

In the Langmuir model, the maximum adsorption capacities (*q*_*m*_) under different conditions were consistent with the kinetic results. For example, *q*_*m*_ values for As(III) adsorption on PS (143.4 mg/kg) and LDPE pellets (98.2 mg/kg) in deionized water were higher than those in river water (112.4 mg/kg and 52.7 mg/kg). The *q*_*m*_ values in this study were significantly lower than those estimated by Dong et al. (2020), who used the smaller sizes of PS particles (0.1–10 µm) for experiments (Table 2). Regarding As(V), the *q*_*m*_ values of PS and LDPE pellets in deionized water were 109.8 mg/kg and 103.5 mg/kg compared to 108.9 mg/kg and 107.7 mg/kg in river water, respectively, which were in accordance with the kinetic results. Moreover, the separation factor (*R*_*L*_) values given in Table 3 are all less than one, indicating that both PS and LDPE pellets had favorable adsorption affinity for As(III) and As(V) (Rawat et al. 2022). From the Freundlich isotherm parameters, all the values of 1/*n* were less than one for PS and LDPE pellets regardless of experimental conditions, suggesting that As(III) and As(V) adsorption on these MPs was non-linear (Table 3). Dong et al. (2020) explained that the interactions between adsorbate and adsorbent caused uneven distribution sites. In that way, the pore space on the adsorbent surface was filled during the adsorption process.

**Table 3:** *R*_*L*_ factor for As(III) and As(V) adsorption on PS and LDPE pellets in deionized water and river water

*R*_*L*_ factor for As(III)				*R*_*L*_ factor for As(V)			
PS-deionized water	LDPE-deionized water	PS-river water	LDPE-river water	PS-deionized water	LDPE-deionized water	PS-river water	LDPE-river water
0.912	0.912	0.859	0.700	0.877	0.899	0.899	0.835
0.828	0.827	0.772	0.548	0.779	0.817	0.803	0.700
0.732	0.729	0.603	0.364	0.664	0.709	0.670	0.532
0.512	0.493	0.357	0.165	0.412	0.440	0.432	0.281
0.380	0.372	0.249	0.108	0.299	0.333	0.311	0.187
0.324	0.317	0.206	0.087	0.247	0.286	0.263	0.153

The three-parameter Sips isotherm model well fitted with experimental data for As(V) in all conditions while only fitted well with As(III) adsorption on PS in deionized water and river water. Moreover, the Sips adsorption capacities (*q*_*m*_) were close to the Langmuir adsorption capacities (*q*_*m*_) when the errors of *q*_*m*_ from Sips equation were less than 10 (mg/kg) (Table 2). The unfavorable As(III) adsorption on LDPE from the Sips model suggested multi-layer adsorption process occurring (Tzabar and ter Brake 2016).

The Langmuir model can describe homogeneous adsorbent surface, while the Freundlich model is suitable for multi-layers of adsorption. Ma et al. (2015) suggested that both types of isotherms can fit the adsorption data well; however, the non-linear Langmuir model described such data better than the non-linear Freundlich model for both As(III) and As(V) (Fig. 3). The results are in agreement with the findings by Ma et al. (2015) who indicated mono-layer As adsorption on sediment which occurred at the specific localized sites and by López-Luna et al. (2019) who found no lateral interaction and steric hindrance between the adsorbed molecules. As a result, As(III) and As(V) adsorbed on selected MPs were suggested to be physisorption, with a higher adsorption capacity of As on PS than on LDPE pellets, except for the adsorption of As(V) in river water.

#### Adsorption isotherms of As(III)/As(V) in river sediments with PS/LDPE pellets

**Fig. 4:**
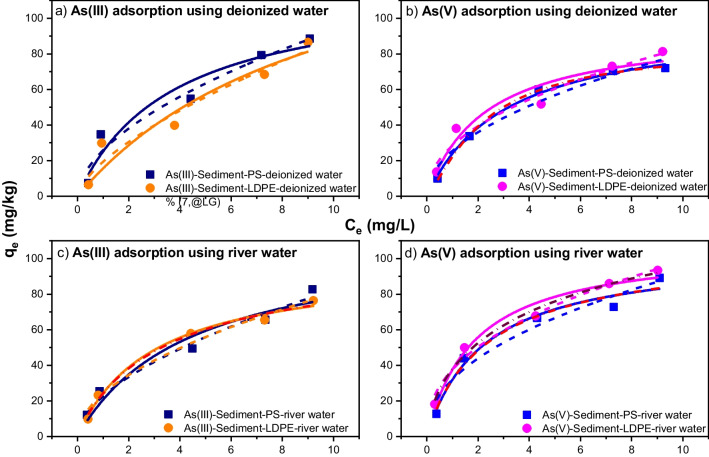
The As(III) adsorption isotherm on sediment-PS pellets and sediment-LDPE pellets (**a** and** c**) and As(V) adsorption isotherm on sediment-PS pellets and sediment-LDPE pellets (**b** and **d**). The solid, dash, and dash dot curves depict results of curve-fitting by the Langmuir, Freundlich, and Sips equations, respectively

The Langmuir and Freundlich isotherm models are used to model the adsorption of As(III) and As(V) by sediment-PS and sediment-LDPE in deionized water and river water, as shown in Fig. 4. According to Zhang and Selim (2005), when the Freundlich parameter (1/*n*) value was much smaller than 1, the adsorption behavior of As was concentration dependent. This parameter was used to measure the extent of the sorption sites’ heterogeneity, providing different adsorption affinities on matrix surfaces for retention. Based on the Freundlich model, the 1/*n* values of 0.53–0.66 for As(III) and 0.42–0.50 for As(V) were found in this study, similar to those obtained by Zhang and Selim (2005) at 0.087–0.368 for As(V) adsorption on different soils, and 0.476–0.556 for As(III) and 0.435–0.625 for As(V) adsorption on lake and river sediments (Ma et al. 2015). The *K*_*F*_ values increased when there were increased amounts of adsorption capacities of both As(III) and As(V), except As(V) adsorbed onto sediment-LDPE in deionized and river water. These results were different from the findings of Zhang and Selim (2005), indicating intensive heterogeneity of adsorption sites for river sediment.

Similar to adsorption kinetics, the Langmuir isotherm model showed lower values of maximum adsorption capacities of sediment in the presence of PS or LDPE than those from the sediment alone in deionized and river water. As reported by Nguyen et al. (2022), the *q*_*m*_ values of sediment for As(III) were 263.3 mg/kg in deionized water and 234.3 mg/kg in river water, while the values for As(V) were 398.7 mg/kg and 206.2 mg/kg in deionized water and river water, respectively. These are significantly higher than those for sediment and MP mixture adsorbents. The *R*_*L*_ factors (Table 4) for the adsorption phase of As(III) and As(V) by the sediment-MP mixtures were less than 1 for all experimental conditions, which indicated that the adsorption process was favorable.

**Table 4:** *R*_*L*_ factor for As(III) and As(V) adsorption by sediment-PS and sediment-LDPE in deionized water and river water

As(III)				As(V)				
Sediment-PS-deionized water	Sediment-LDPE-deionized water	Sediment-PS-river water	Sediment-LDPE-river water		Sediment-PS-deionized water	Sediment-LDPE-deionized water	Sediment-PS-river water	Sediment-LDPE-river water
0.899	0.959	0.922	0.878		0.881	0.856	0.857	0.858
0.805	0.913	0.837	0.787		0.653	0.666	0.608	0.559
0.459	0.724	0.497	0.403		0.419	0.339	0.342	0.305
0.343	0.577	0.376	0.290		0.302	0.240	0.235	0.208
0.292	0.526	0.326	0.245		0.252	0.199	0.198	0.172

The Sips model well fitted with As(V) adsorption in sediment-PS-deionized water, sediment-PS-river water, and sediment-LDPE-river water conditions as well as As(III) adsorption in sediment-LDPE-river water (Table 2). The Sips adsorption capacities for these results were close to the estimated Langmuir adsorption capacities, except for As(V) adsorption in sediment-LDPE-river water, with high value of Sips *q*_*m*_ error (171.9 ± 104.8 mg/kg). In other conditions, the experimental data were unable to be modeled by the Sips model which describes only mono-layer adsorption systems (Tzabar and ter Brake 2016), while the adsorption process may occur in multi-layer fashion.

### Adsorption thermodynamics

All experiments were conducted at laboratory temperature (20 °C); therefore, the entropy change (Δ*S*^o^) and enthalpy change (Δ*H*^o^) parameters in thermodynamics were unable to be calculated. In this study, only Gibbs free energy change (ΔG) is shown in Table 5. The negative values of Δ*G* in all experimental conditions indicate that As(III) and As(V) adsorption by MP pellets (PS and LDPE) and a combination of these MPs with the river sediment is spontaneous.

**Table 5:** Thermodynamic Gibbs free energy change (Δ*G*) parameter of As(III) and As(V) adsorption

Adsorbents and solutions	*K*_*e*_ (L/kg)		Δ*G* (kJ/kg)	
	As(III)	As(V)	As(III)	As(V)
PS-deionized water	10.41011	8.899843	− 331.123	− 308.97
LDPE-deionized water	7.867654	8.465103	− 291.546	− 301.891
PS-river water	9.9839	8.576242	− 325.215	− 303.735
LDPE-river water	5.1934	10.33739	− 232.839	− 330.133
Sediment-PS-deionized water	9.76033	7.727117	− 322.014	− 288.999
Sediment-LDPE-deionized water	9.601626	8.81339	− 319.697	− 307.59
Sediment-PS-river water	9.009504	9.799254	− 310.701	− 322.577
Sediment-LDPE-river water	8.282978	10.34031	− 298.817	− 330.173

### FTIR results of PS, LDPE and sediment

#### FTIR results of PS and LDPE pellets

**Fig. 5:**
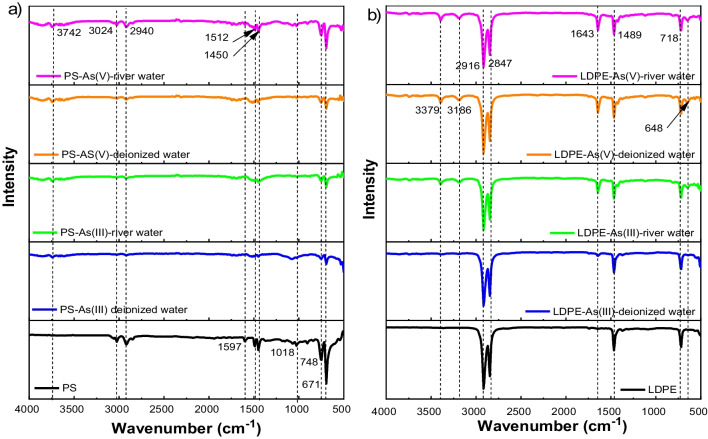
FTIR spectra of PS pellets (**a**) and LDPE pellets (**b**) before and after As(III)/As(V) adsorption

Figure 5 presents the FTIR spectra results of PS and LDPE pellets before and after interactions with As(III) and As(V), in deionized and river water. As can be seen from IR spectra of PS (Fig. 5a), the IR adsorption peak at 3024 cm^−1^ disappeared after As(III) adsorption in deionized water, however, two new peaks of 3742 cm^−1^ and 3842–3858 cm^−1^ appeared after the adsorption of both As(III) and As(V). The peaks of over 3000 cm^−1^ were attributed to O–H stretching vibrations (Dong et al. 2020). The IR peaks at 1443–1697 cm^−1^ characterized the amide C=$\mathrm{O}$ stretching (Dong et al. 2020; Rawat et al. 2022). In this functional group, the peak of 1597 cm^−1^ before the adsorption on PS disappeared after the adsorption of As(III) under deionized water, while it shifted to 1690 cm^−1^ after the adsorption of As(V) in deionized water, and to 1697 cm^−1^ after the adsorption of As(III) and As(V) in river water. The IR bands at 1497 cm^−1^ and 1443 cm^−1^ were stable in river water, but shifted to 1512 cm^−1^ and 1450 cm^−1^ in deionized water for both As(III) and As(V). Moreover, the IR band at 1018 cm^−1^ before adsorption, which slightly shifted to 1080 cm^−1^ after adsorption of As(III) in deionized water, disappeared after the adsorption of As(V) in deionized water, and divided into two peaks (1022 and 1026 cm^−1^) after the adsorption of As(V) in river water, attributed to O–H bend of PS (LibreTexts 2021). According to Dong et al. (2020), the peak at 748 cm^−1^, which shifted to 756 cm^−1^ after As(III) and As(V) adsorption, can be attributed to the long chain CH_2_. Generally, two peaks were involved in As(III) binding with PS pellets, while the new peaks indicated strong surface interaction and formation of new bonds between As(III) or As(V) and surface functional groups of hydroxyl and carboxyl.

The FTIR spectra results for surface functional groups of LDPE related to the adsorption of As(III) and As(V) are shown in Fig. 5b. According to Chen et al. (2001), the peaks of 2909 cm^−1^ and 2847 cm^−1^ correspond to strong C–H asymmetric stretching and medium strong C–H symmetric stretching, of which the band 2909 cm^−1^ slightly shifted to 2916 cm^−1^ after adsorption of As(V) in both deionized water and river water, while the later peak was stable after adsorption regardless of conditions. Two new bands of 3379–3395 cm^−1^ and 3186–3194 cm^−1^ appeared after As(III) and As(V) adsorption, indicating that new bonds between As(III) or As(V) and surface hydroxyl functional group were being formed. Additionally, two other new bands at 1643 cm^−1^ and 648 cm^−1^ were assigned to the amide C = O stretching and O–H bend functional groups (Dong et al. 2020; LibreTexts 2021). As a result, although there were differences among the values of IR peaks of LDPS and PS, they shared similar surface functional groups for the interactions with As(III) and As(V).

#### FTIR results of PS and LDPE in the presence of sediment

**Fig. 6:**
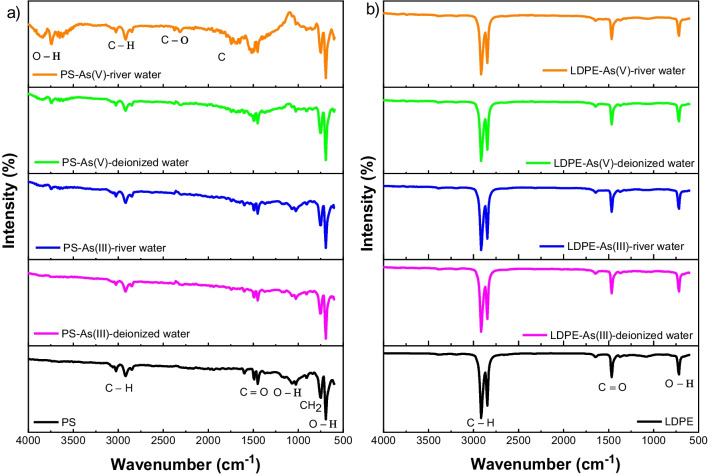
FTIR spectra of PS pellets (**a**) and LDPE pellets (**b**) before and after As(III) and As(V) adsorption in the presence of sediment with deionized water and river water

Figure 6 illustrates the FTIR spectra results of PS and LDPE pellets before and after interactions with As(III) and As(V), in the presence of sediment in both deionized water and river water solutions. The FTIR results of PS beads before and after As(III) and As(V) adsorption in deionized water and river water are shown in Fig. 6a. The peaks at 748 and 2847 cm^−1^ slightly shifted to 756 and 2855 cm^−1^ after the adsorption of As(III) in both deionized water and river water, which were assigned to the presence of CH_2_ chain (Dong et al. 2020). Regarding As(V) species, FTIR peaks were changed after the adsorption of As(V) in river water. A new band at 3742 cm^−1^ was assigned to O–H functional groups, while the peaks of 1681–1744 cm^−1^, 2315 cm^−1^, and 2855 cm^−1^ were associated with C–N, C–O, and O–H groups, respectively (Dong et al. 2020; Misra et al. 2006). Three main functional groups including C–H, C = O, and O–H before and after As(III) and As(V) adsorption can be seen at LDPE surface (Fig. 6b). There was a slight change for the peak of 3194 cm^−1^ before the adsorption which shifted to 3186 cm^−1^ after the adsorption of As(III) and As(V), which was attributed to the involvement of C–H functional group in the binding of As species. There was a significant difference for the peaks around 1450–1512 cm^−1^ related to C-N vibration of PS (sediment-PS) compared to two peaks at 1450 cm^−1^ and 1512 cm^−1^ of PS only after the adsorption of As(V) in river water. For the LDPE pellets, the appearance of a peak at 1643 cm^−1^ after the adsorption of As(III) in river water and As(V) in both deionized water and river water was not found in sediment-LDPE system.

#### FTIR results of sediment in the presence of PS or LDPE

**Fig. 7:**
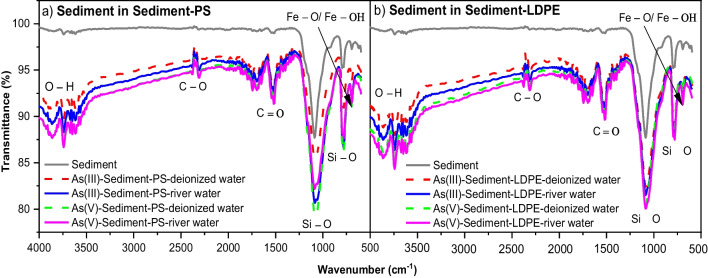
FTIR spectra of river sediment before and after adsorption of As(III) and As(V) in deionized and river water, in the presence of **a** PS and **b** LDPE pellets

Figure 7 presents the FTIR spectra results of the river sediment before and after interactions with As(III) and As(V), in the presence of PS or LDPE in deionized water and river water. Compared to the surface of river sediment particles before adsorption, the main functional groups involved in the interactions with As species were phenolic hydroxyl, carboxyl, quartz, and goethite groups. In the phenolic hydroxyl group, a new band of 3865 cm^−1^ appeared after As adsorption. The new peak at 2307 cm^−1^ was attributed to C–O molecular vibrations in calcite (Hahn et al. 2018). In the carboxyl group, two new peaks of 1651 cm^−1^ and 1705 cm^−1^ were assigned to amine C = O stretching (Yu et al. 2015). Moreover, the FTIR bands at 795 cm^−1^ and 1088 cm^−1^ were attributed to Si–O symmetrical stretching vibrations and Si(Al)–O vibration or antisymmetric stretching vibrations of Si–O tetrahedron of quartz (Hahn et al. 2018), while the peak of 694 cm^−1^ was associated with Fe–O/Fe–OH vibration of the magnetite phase (Luo et al. 2012; Rawat et al. 2022).

### Adsorption mechanisms of As(III)/As(V) by PS, LDPE, and sediment

For the adsorption of As by MP particles, the binding sites on the adsorbent surface were supported by FTIR results (Fig. 5). It was reported that the H atoms on the carboxyl group for PS and hydroxyl group for PTFE have large positive electrostatic potential of + 56.60 and + 82.37 kcal/mol, respectively (Dong et al. 2020, 2019). The O–H bond length was shortened due to the O atom participating in the complexation of As(III) or As(V) during the adsorption process (Dong et al. 2020). This study revealed that adsorption of As metal ion is related to nitrogen and oxygen functional groups of adsorbent surface (Gordon et al. 2015). Based on the surface electrostatic potential analysis of PS and PTFE (Dong et al. 2020, 2019), the key mechanisms for As(III) adsorption onto PS and PTFE are electrostatic force and non-covalent interaction. Sharing similar FTIR results with Dong et al. (2020) for PS adsorbed As(III), the results suggested that electrostatic force and non-covalent interaction are the main factors affecting adsorption mechanism of As(III) and As(V) on PS and LDPE.

For As adsorption on sediment, the FTIR results (Fig. 7) of river sediment particles after As(III) and As(V) adsorption illustrated that several types of surface functional groups participated in the interactions between As(III) or As(V) with sediment. The new peaks of 1651, 1705, 2307, and 3865 cm^−1^ on the surface of sediment associated with As(III) or As(V) are related to amine C = O stretching, C–O molecular vibrations and phenolic hydroxyl functional groups (Hahn et al. 2018; Yu et al. 2015). According to Rawat et al. (2022), As(V) can interact with organic-Si or with SiO_2_ in the presence of organic matter to create the As–O–Si form. In addition, the carboxyl group was involved in binding inorganic As(V) via sediment organic matter (Rawat et al. 2022). The presence of Fe–O surface groups in As(III) and As(V) adsorption possibly facilitated As(V) immobilization by bidentate complex (-Fe_2_HAsO_4_^2−^) or monodentate complex (-FeH_2_AsO_4_^−^) (Rawat et al. 2022; Sun et al. 2009). This process occurred via surface complexation or coordination (Sun et al. 2018) and transformation from As(III) to As(V). There was no clear difference in the functional groups of river sediment after As(III) and As(V) adsorption, suggesting that As(V) and As(III) may share similar adsorption mechanisms in their interactions with sediment.

In addition, the surface functional groups of PS pellets and LDPE pellets also contributed to the interactions with As(III) and As(V) during the adsorption process (Fig. 6). The FTIR results for PS pellets after adsorption of As(V) revealed that -NH_2_ and -OH functional groups of an adsorbent participated in the adsorption of metal ions (Darnall et al. 1986). Regarding As(III), Dong et al. (2020) reported that As(III) adsorbed on PS pellets via chemisorption, in which hydrogen bonds were produced when trivalent arsenic interacted with carboxyl group. The FTIR results for LDPE pellets revealed that As(III) and As(V) adsorption occurred on the functional groups of -COOH and -OH (Irani et al. 2015). These findings indicated that electrostatic attraction and chelation were the physisorption and complexation mechanisms for metal ion adsorption beside ion exchange (Irani et al. 2015).

## Conclusions

In this study, the roles of PS and LDPE in the adsorptive behavior of As(III) and As(V) in river environment were investigated. The adsorption kinetics of As(III) and As(V) on PS and LDPE were modeled well by the PFO and PSO models. PS showed a higher adsorption capacity than LDPE for As, except for As(V) adsorption in river water. The Elovich kinetic model shows that most adsorption processes of As(III) and As(V) by MP particles (PS and LDPE) or combined with the river sediment in deionized water and river water occurred at the mild rising zone of naturally chemical adsorption, except the adsorption of As(V) on sediment-PS-river water and sediment-LDPE-river water. The results showed that both MP characteristics and water properties affected the adsorption of inorganic As species. The adsorption equilibrium of As(III) and As(V) followed both Langmuir and Freundlich isotherm models. The Langmuir model showed a higher adsorption capacity (*q*_*m*_) of As(III) than As(V) by PS, in contrast to a higher adsorption of As(V) than As(III) by LDPE. The Sips isotherm model partly fitted with the experimental data, suggesting that some experimental processes occurred in multi-layer mode. The negative values of Gibbs free energy change (Δ*G*) of the adsorption thermodynamics indicate the spontaneous adsorption process for both As(III) and As(V). The FTIR examination demonstrated that the surface complexation or coordination of As(III)/As(V) with sediment surface functional groups was the main adsorption mechanism. The interactions of As species with PS mainly occurred via -NH_2_ and -OH functional groups, while -COO- and -OH functional groups contributed to the adsorption mechanism of As species on LDPE. Overall, river sediment was found to be most effective in the adsorption of As than MP particles.

## Data Availability

The datasets used and/or analyzed during the current study are available from the corresponding author on reasonable request.
